# Virus altered rice attractiveness to planthoppers is mediated by volatiles and related to virus titre and expression of defence and volatile-biosynthesis genes

**DOI:** 10.1038/srep38581

**Published:** 2016-12-07

**Authors:** Guanghua Lu, Tong Zhang, Yuange He, Guohui Zhou

**Affiliations:** 1 Guangdong Province Key Laboratory of Microbial Signals and Disease Control, College of Agriculture, South China Agricultural University, Guangzhou, Guangdong 510642, China

## Abstract

Viruses may induce changes in plant hosts and vectors to enhance their transmission. The white-backed planthopper (WBPH) and brown planthopper (BPH) are vectors of *Southern rice black-streaked dwarf virus* (SRBSDV) and *Rice ragged stunt virus* (RRSV), respectively, which cause serious rice diseases. We herein describe the effects of SRBSDV and RRSV infections on host-selection behaviour of vector and non-vector planthoppers at different disease stages. The Y-tube olfactometer choice and free-choice tests indicated that SRBSDV and RRSV infections altered the attractiveness of rice plants to vector and non-vector planthoppers. The attractiveness was mainly mediated by rice volatiles, and varied with disease progression. The attractiveness of the SRBSDV- or RRSV-infected rice plants to the virus-free WBPHs or BPHs initially decreased, then increased, and finally decreased again. For the viruliferous WBPHs and BPHs, SRBSDV or RRSV infection increased the attractiveness of plants more for the non-vector than for the vector planthoppers. Furthermore, we observed that the attractiveness of infected plants to planthoppers was positively correlated with the virus titres. The titre effects were greater for virus-free than for viruliferous planthoppers. Down-regulated defence genes *OsAOS1, OsICS*, and *OsACS2* and up-regulated volatile-biosynthesis genes *OsLIS, OsCAS*, and *OsHPL3* expression in infected plants may influence their attractiveness.

Plant–pathogen–vector insect ecosystems are characterized by complex direct and indirect interactions^1^,^2^, which may influence the host-selection behaviour of vectors to enhance pathogen transmission^3^,^4^,^5^,^6^. Vector or non-vector insect preference behaviour (i.e., vector attraction, selection, settling, feeding, and other parameters) is influenced by the pathogen infecting the host plants, which are often preferentially oriented^1^,^7^,^8^,^9^,^10^,^11^. In the plant mediated indirect interaction between virus and its insect vector, the virus could induce plant defence responses which influence the host–vector interactions by modifying the phenotypes of the host plants, like activating relevant signalling pathways, including jasmonic acid (JA), salicylic acid (SA) and ethylene (ET)^12^,^13^,^14^. Simultaneously, plants could also regulate the release of volatile organic compounds induced by viruses, which can be used by vector insects to locate their host, which may enhance the spread of the virus^1^,^15^,^16^,^17^,^18^,^19^. The long-distance migrating white-backed planthopper (WBPH; *Sogatella furcifera*) and the brown planthopper (BPH; *Nilaparvata lugens*) are two of the most destructive rice pests. They severely damage plants by removing the phloem sap. They also transmit plant viruses, including *Southern rice black-streaked dwarf virus* (SRBSDV) by WBPH and *Rice ragged stunt virus* (RRSV) by BPH.

*Southern rice black-streaked dwarf virus* is a member of the genus *Fijivirus* in the family Reoviridae. Plants infected by SRBSDV exhibit stunted growth and produce darkened leaves as well as small enations on the stem and the underside of leaves^20^. This virus was first detected in Guangdong, China in 2001 as the causative agent of a new rice disease called southern rice black-streaked dwarf disease^20^. In 2011 and 2012, the disease-affected areas in China and Vietnam were 700,000 and 500,000 hectares, respectively^21^,^22^, and the virus has also been observed in Japan^23^. SRBSDV is efficiently transmitted by WBPH in a circulative, propagative, and persistent manner, but cannot be transmitted by BPH or by eggs or rice seeds^20^,^24^. Adult WBPHs transmit SRBSDV to rice seedlings in newly colonized areas and lay eggs on host plants during their migration. Because of the dynamic migration patterns of WBPH, it is very difficult to control outbreaks of southern rice black-streaked dwarf disease and infestations of insect pests on agricultural land.

*Rice ragged stunt virus* is a member of the genus *Oryzavirus* in the family Reoviridae, and causes rice ragged stunt disease. It was first described in 1978, and is distributed in Indonesia and the Philippines^25^. It subsequently became prevalent in most rice-growing regions in Southeast Asia and southern China^26^,^27^. Following its discovery, RRSV has become one of the most important rice pathogens in these regions. Infections by RRSV have recently started occurring again, with BPH as a vector, in the same manner as infections caused by SRBSDV^28^. BPH acquire the virus by ingesting the sap of RRSV-infected rice plants and transmit it to other plants. Rice plants are commonly simultaneously infected by RRSV and SRBSDV, and the co-infection leads to increased viruliferous rates for WBPHs and BPHs, resulting in enhanced spread of SRBSDV and RRSV^29^.

We recently revealed that SRBSDV-infected rice plants are more attractive to virus-free WBPHs and RRSV-carrying BPHs, but less attractive to viruliferous WBPHs and virus-free BPHs, compared with uninfected plants^6^. Nevertheless, lack of a comprehensive and integrated characterization of host physiology and vector behaviour after viral infections has limited our understanding of the evolutionary significance of plant–virus interactions as well as the underlying mechanisms. The objective of this study was to clarify the relationship between the attraction of rice planthoppers to SRBSDV- or RRSV-infected rice plants and disease progression. We also aimed to identify the major factors influencing the dynamics of the attraction of vector and non-vector rice planthoppers to virus-infected plants. We compared the preferences of rice planthoppers in the Y-tube olfactometer choice and free-choice assays at different rice disease stages. Additionally, we analysed the relative chlorophyll contents, virus titres, and the expression levels of rice genes related to defence (*OsAOS1, OsICS*, and *OsACS2*) and volatile biosynthesis (*OsLIS, OsCAS*, and *OsHPL3*). Our study may have important implications for clarifying the interactions in the ‘SRBSDV or RRSV–WBPH or BPH–rice’ systems and predicting the epidemiological characteristics of these viruses under field conditions.

## Results

### Attractiveness of SRBSDV-infected plants to planthoppers in the Y-tube olfactometer choice test

**Figure 1:**
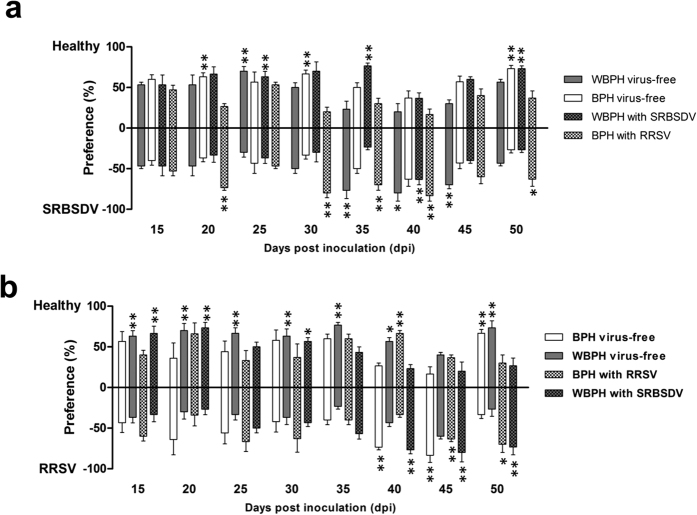
Behavioural responses of white-backed planthoppers (WBPH) and brown planthoppers (BPH) to volatile cues from healthy and virus-infected plants. In pair-wise choice tests conducted using a Y-tube olfactometer, SRBSDV-infected (**a**) and RRSV-infected (**b**) rice plants were compared with healthy plants during disease progression. Virus-free and viruliferous planthoppers were evaluated separately. Data were analysed by generalized linear models and are presented as the mean ± standard error. Data are arranged vertically by the pairs involved in each choice test. *p < 0.05 and **p < 0.01 for pair-wise comparisons.

Planthopper behavioural responses to volatile cues from healthy and SRBSDV-infected rice plants were tested by pair-wise choice tests using a Y-tube olfactometer. Compared with that of healthy rice plants, the attractiveness of SRBSDV-infected rice plants to the virus-free vector WBPHs significantly decreased at 25 days post infection (dpi) (30.0%, t = 4.899, df = 4, p = 0.008), significantly increased at 35, 40, and 45 dpi (76.7%, t = 11.314, df = 4, p < 0.001; 80%, t = 4.243, df = 4, p = 0.013; 70%, t = 4.899, df = 4, p = 0.008, respectively), and gradually decreased at 50 dpi (Fig. 1a). The virus-free non-vector BPHs were least attracted to SRBSDV-infected rice plants at 20 and 30 dpi (36.7%, t = 5.657, df = 4, p = 0.005; 33.3%, t = 7.071, df = 4, p = 0.002, respectively), but the attraction slightly increased at 40 dpi and significantly decreased at 50 dpi (26.7%, t = 9.899, df = 4, p = 0.001) (Fig. 1a). For the viruliferous planthoppers, the attractiveness of SRBSDV-infected rice plants to SRBSDV-carrying WBPHs obviously decreased at 25 and 35 dpi (36.7%, t = 5.657, df = 4, p = 0.005; 36.7%, t = 4.950, df = 4, p = 0.008, respectively), significantly increased at 40 dpi (63.3%, t = 5.657, df = 4, p = 0.005), and significantly decreased at 50 dpi (26.7%, t = 9.899, df = 4, p = 0.001) (Fig. 1a). In contrast, the attractiveness of SRBSDV-infected rice plants to RRSV-carrying BPHs increased at most time points, with significant increases at 20, 30, 35, 40, and 50 dpi (73.3%, t = 10.744, df = 4, p < 0.001; 80.0%, t = 7.348, df = 4, p = 0.002; 70.0%, t = 4.899, df = 4, p = 0.008; 83.3%, t = 7.071, df = 4, p = 0.002; 63.3%, t = 5.657, df = 4, p = 0.005, respectively).

### Attractiveness of RRSV-infected plants to planthoppers in the Y-tube olfactometer choice test

We analysed the effects of volatiles emitted from RRSV-infected rice plants on the preferences of planthoppers. Compared with that of healthy rice plants, the attractiveness of RRSV-infected rice plants to the virus-free vector BPHs slightly decreased at 15–35 dpi, significantly increased at 40 and 45 dpi (73.3%, t = 9.899, df = 4, p = 0.001; 83.3%, t = 5.345, df = 4, p = 0.006, respectively), and then significantly decreased at 50 dpi (33.3%, t = 7.071, df = 4, p = 0.002) (Fig. 1b). For the virus-free non-vector WBPHs, the attractiveness of RRSV-infected rice plants evidently decreased at 15–40 dpi, slightly increased at 45 dpi (60%, t = 2.449, df = 4, p = 0.070), and significantly decreased at 50 dpi (Fig. 1b). For the viruliferous planthoppers, the attractiveness of RRSV-infected rice plants to RRSV-carrying BPHs significantly decreased at 40 dpi (33.3%, t = 7.071, df = 4, p = 0.002) and significantly increased at 45 and 50 dpi (63.3%, t = 5.657, df = 4, p = 0.005; 70%, t = 2.828, df = 4, p = 0.047, respectively) (Fig. 1b). The attractiveness of RRSV-infected rice plants to SRBSDV-carrying WBPHs dramatically decreased at 15, 20, and 30 dpi (33.3%, t = 7.071, df = 4, p = 0.002; 26.7%, t = 9.899, df = 4, p = 0.001; 43.3%, t = 2.828, df = 4, p = 0.047, respectively) and significantly increased at 40, 45, and 50 dpi (76.7%, t = 11.314, df = 4, p < 0.001; 80%, t = 4.243, df = 4, p = 0.013; 73.3%, t = 4.950, df = 4, p = 0.008, respectively) (Fig. 1b).

### Attractiveness of SRBSDV-infected plants to planthoppers in the free-choice test

**Figure 2:**
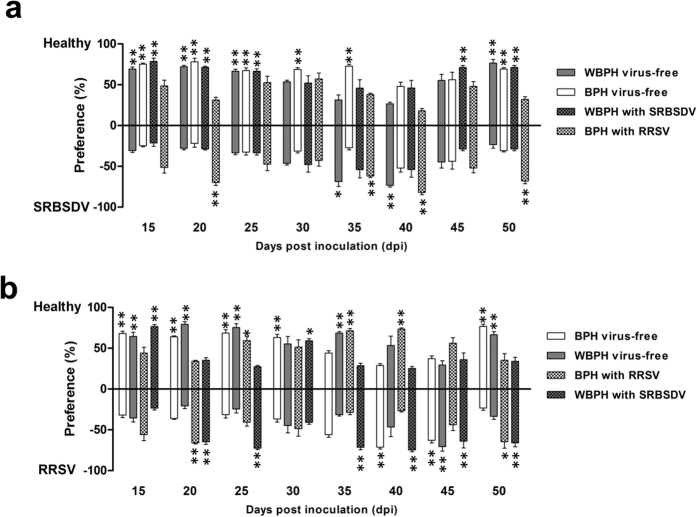
Free choice of white-backed planthoppers (WBPH) and brown planthoppers (BPH) for healthy and virus-infected plants. In pair-wise free-choice tests, SRBSDV-infected (**a**) and RRSV-infected (**b**) rice plants were compared with healthy plants during disease progression. Virus-free and viruliferous planthoppers were evaluated separately. Data were analysed by generalized linear models and are presented as the mean ± standard error. Data are arranged vertically by the pairs involved in each choice test. *p < 0.05 and **p < 0.01 for pair-wise comparisons.

We next investigated planthopper preferences using a free-choice test involving all plant cues. Compared with that of healthy rice plants, the attractiveness of SRBSDV-infected rice plants to virus-free vector WBPHs significantly decreased at 15–25 dpi and increased at 35 and 40 dpi (68.6%, t = 13.238, df = 8, p < 0.001; 73.4%, t = 7.176, df = 8, p < 0.001, respectively), and significantly decreased at 50 dpi (23.3%, t = 9.346, df = 8, p = 0.002) (Fig. 2a). For the virus-free non-vector BPHs, the attractiveness of SRBSDV-infected rice plants obviously decreased at 15, 20, 25, 30, and 35 dpi (24.7%, t = 10.256, df = 8, p = 0.003; 21.9%, t = 7.372, df = 8, p = 0.001; 32.7%, t = 11.354, df = 8, p < 0.001; 31.3%, t = 8.416, df = 8, p = 0.007; 37.3%, t = 10.792, df = 8, p < 0.001, respectively), slightly increased at 40 and 45 dpi, and significantly decreased at 50 dpi (30.7%, t = 7.230, df = 8, p < 0.001) (Fig. 2a). For the viruliferous planthoppers, the attractiveness of SRBSDV-infected rice plants to SRBSDV-carrying WBPHs significantly decreased at 15, 20, and 25 dpi (21.3%, t = 6.783, df = 8, p = 0.015; 28.7%, t = 10.694, df = 8, p < 0.001; 33.3%, t = 8.286, df = 8, p = 0.023, respectively), slightly increased at 30–40 dpi, and significantly decreased at 45 and 50 dpi (71.3%, t = 8.784, df = 8, p = 0.003; 72%, t = 10.250, df = 8, p = 0.003, respectively) (Fig. 2a). For the RRSV-carrying BPHs, the attractiveness of SRBSDV-infected rice plants evidently increased at 20, 35, 40, and 50 dpi (70%, t = 7.562, df = 8, p = 0.014; 62%, t = 11.064, df = 8, p < 0.001; 82%, t = 9.638, df = 8, p = 0.02; 68%, t = 10.732, df = 8, p < 0.001, respectively). The RRSV-carrying BPHs exhibited no preference differences at all other time points (Fig. 2a).

### Attractiveness of RRSV-infected plants to planthoppers in the free-choice test

We analysed the preferences of planthoppers freely able to choose between RRSV-infected and healthy rice plants. Compared with that of healthy plants, the attractiveness of RRSV-infected rice plants to virus-free vector BPHs significantly decreased at 15–30 dpi, significantly increased at 40 and 45 dpi (71.3%, t = 13.296, df = 8, p < 0.001; 62.7%, t = 5.539, df = 8, p = 0.001, respectively), and significantly decreased at 50 dpi (23.3%, t = 11.036, df = 8, p < 0.001) (Fig. 2b). For the virus-free non-vector WBPHs, the attractiveness of RRSV-infected rice plants significantly decreased at 15, 20, 25, and 35 dpi (35.3%, t = 7.736, df = 8, p = 0.003; 20.7%, t = 9.256, df = 8, p = 0.012; 24.7%, t = 10.318, df = 8, p = 0.007; 31.3%, t = 7.436, df = 8, p < 0.001, respectively), significantly increased at 45 dpi (70.7%, t = 7.588, df = 8, p < 0.001), and significantly decreased at 50 dpi (33.4%, t = 9.054, df = 8, p = 0.013) (Fig. 2b). For the viruliferous planthoppers, the attractiveness of RRSV-infected rice plants to RRSV-carrying BPHs obviously increased at 20 and 50 dpi (66.0%, t = 18.013, df = 8, p < 0.001; 64.7%, t = 2.590, df = 8, p = 0.032, respectively) and significantly decreased at 25, 35, and 40 dpi (40.7%, t = 2.646, df = 8, p = 0.029; 28.7%, t = 11.114, df = 8, p < 0.001; 26.7%, t = 22.032, df = 8, p < 0.001, respectively) (Fig. 2b). The attractiveness of RRSV-infected rice plants to SRBSDV-carrying WBPHs significantly decreased at 15 and 30 dpi (76.7%, t = 16.012, df = 8, p < 0.001; 59.3%, t = 5.369, df = 8, p = 0.001, respectively) and significantly increased at 20, 25, 35, 40, 45, and 50 dpi (64.7%, t = 10.242, df = 8, p = 0.001; 72.7%, t = 9.372, df = 8, p = 0.003; 71.3%, t = 9.184, df = 8, p = 0.003; 74.7%, t = 11.722, df = 8, p < 0.001; 64%, t = 9.968, df = 8, p = 0.001; 66%, t = 10.052, df = 8, p < 0.001, respectively) (Fig. 2b).

A comparison of the results of the two assays revealed that the host-selection preferences of the planthoppers were similar between the two tests. These findings suggest that the virus-induced changes to the preferences of planthoppers for infected rice plants are mainly mediated by rice volatiles.

### Relative chlorophyll content was not correlated with rice plant attractiveness

**Figure 3:**
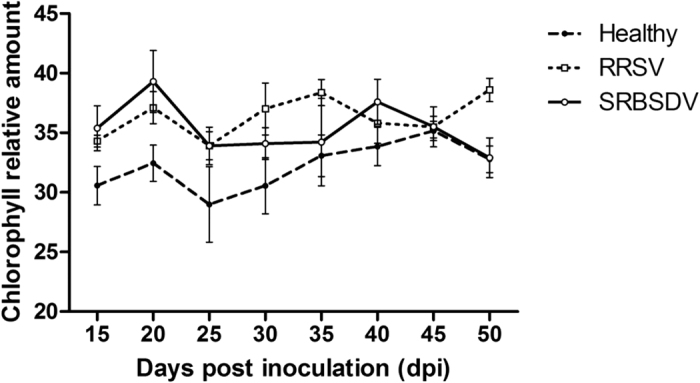
Time course of chlorophyll accumulation in the leaves of healthy and virus-infected rice plants. The chlorophyll contents of fully expanded leaves were analysed using a chlorophyll meter at the indicated times. Each data point represents the mean ± standard deviation of six biological replicates.

The relative chlorophyll contents of SRBSDV- or RRSV-infected plants and healthy controls were analysed. At 15–50 dpi, the relative chlorophyll content of SRBSDV-infected rice plants was higher than that of healthy plants. The differences were significant at 40 dpi (t = 2.439, df = 10, p = 0.035) (Fig. 3). The chlorophyll content trends in SRBSDV-infected plants were not correlated with their attractiveness to virus-free vector WBPHs (Pearson, r = 0.465, p = 0.141), virus-free non-vector BPHs (Pearson, r = 0.382, p = 0.168), SRBSDV-carrying WBPHs (Pearson, r = 0.532, p = 0.257), and RRSV-carrying BPHs (Pearson, r = 0.375, p = 0.621). Similarly, the relative chlorophyll contents of RRSV-infected plants were higher than those of healthy plants, with significant differences observed at 15, 20, 25, and 50 dpi (t = 3.815, df = 10, p = 0.003; t = 4.251, df = 10, p = 0.002; t = 2.538, df = 10, p = 0.029; t = 2.582, df = 10, p = 0.027, respectively). The relative chlorophyll content trends in RRSV-infected plants were also not correlated with the attractiveness of plants to virus-free vector BPHs (Pearson, r = 0.505, p = 0.087), virus-free non-vector WBPHs (Pearson, r = 0.447, p = 0.237), RRSV-carrying BPHs (Pearson, r = 0.494, p = 0.528), and SRBSDV-carrying WBPHs (Pearson, r = 0.332, p = 0.075).

### Virus titre was correlated with rice plant attractiveness

**Figure 4:**
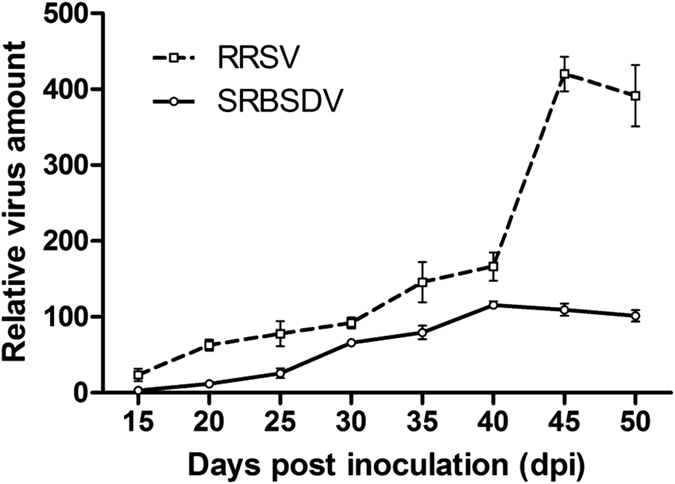
Time course of relative viral genome accumulation in the leaves of infected rice plants. Total RNA was extracted from leaf sheaths collected at the indicated times. Samples were analysed for viral genome accumulation by quantitative reverse transcription polymerase chain reaction. Primers specific for genome segment 10 of SRBSDV and genome segment 8 for RRSV were used to quantify the relative viral genome titre. The gene encoding the rice U6 small nuclear RNA was used as an internal standard. Each data point represents the mean ± standard deviation of three biological replicates.

The SRBSDV and RRSV titres during infection were quantified by quantitative reverse transcription polymerase chain reaction (RT-qPCR). For the SRBSDV-infected plants, the virus titre gradually increased at 15–40 dpi, reaching the maximum level at 40 dpi, and then slowly declined at 40–50 dpi (Fig. 4). The virus titre was positively linearly correlated with the attractiveness of host plants to virus-free vector WBPHs (Pearson, r = 0.835, p = 0.034) and virus-free non-vector BPHs (Pearson, r = 0.645, p = 0.084). However, it was not correlated with the attractiveness of plants to SRBSDV-carrying WBPHs (Pearson, r = 0.557, p = 0.151) or RRSV-carrying BPHs (Pearson, r = 0.345, p = 0.402). For the RRSV-infected plants, the virus titre gradually increased at 15–45 dpi, reaching the maximum level at 45 dpi, and moderately declined at 45–50 dpi. The virus titre was positively linearly correlated with the attractiveness of plants to virus-free vector BPHs (Pearson, r = 0.732, p = 0.047) and virus-free non-vector WBPHs (Pearson, r = 0.681, p = 0.063). In contrast, it was not correlated with the attractiveness of plants to RRSV-carrying BPHs (Pearson, r = 0.058, p = 0.852) or SRBSDV-carrying WBPHs (Pearson, r = 0.403, p = 0.322). These results indicate that SRBSDV and RRSV infections influence the attractiveness of host plants to virus-free vector or non-vector planthoppers.

### Expression of rice defence-related genes was affected by viruses

**Figure 5:**
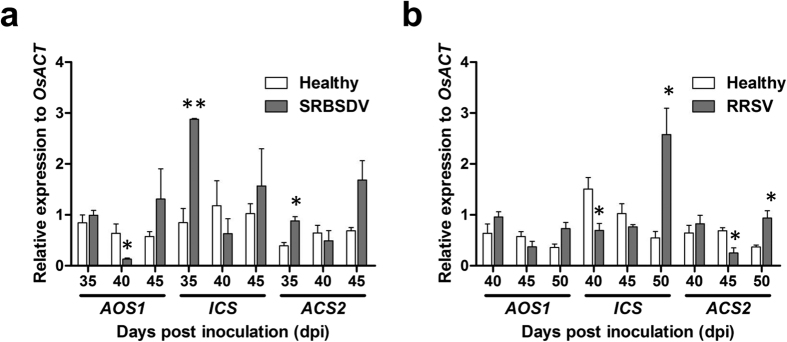
Effect of viral infections on rice defence responses. The transcript levels of jasmonic acid (*AOS1*), salicylic acid (*ICS*), and ethylene (*ACS2*) defence genes in SRBSDV-infected (**a**) and RRSV-infected (**b**) plants were compared with those of healthy plants. Total RNA was extracted from leaf sheaths collected at the indicated times and analysed by quantitative reverse transcription polymerase chain reaction. *OsACT* was used as an internal standard. Each data point represents the mean ± standard deviation of three biological replicates. *p < 0.05 and **p < 0.01 for comparisons with healthy plants.

To determine whether SRBSDV or RRSV influences plant defence responses, we compared the expression levels of key genes involving JA, SA, and ET biosynthesis in healthy, SRBSDV-infected, and RRSV-infected rice plants at the time points at which the viral titres were highest. The *OsICS* (SA biosynthesis gene) and *OsACS2* (ethylene biosynthesis gene) transcript abundances were higher in the SRBSDV-infected plants than in the healthy controls at 35 dpi (t = 7.310, df = 4, p = 0.002; t = 4.566, df = 4, p = 0.010, respectively) (Fig. 5a). The expression level of *OsAOS1* (JA biosynthesis gene) was approximately 80% lower in the SRBSDV-infected plants than in the healthy controls at 40 dpi (t = 2.777, df = 4, p = 0.05) (Fig. 5a).

There were no differences in the *OsAOS1* transcript levels between RRSV-infected and healthy plants at all three analysed time points (Fig. 5b). However, the *OsICS* expression level was about 50% lower at 40 dpi (t = 3.095, df = 4, p = 0.036) and about 3-fold higher at 50 dpi (t = 3.822, df = 4, p = 0.019) in the RRSV-infected plants than in the healthy plants (Fig. 5b). The *OsACS2* transcript abundance was about 50% lower at 45 dpi (t = 3.178, df = 4, p = 0.034) and about 2.5-fold higher at 50 dpi (t = 3.955, df = 4, p = 0.017) in the RRSV-infected rice plants than in the healthy plants (Fig. 5b). These results provide a general overview of how SRBSDV and RRSV affect rice defence responses.

### Expression of rice volatile-biosynthesis genes was induced by viruses

**Figure 6:**
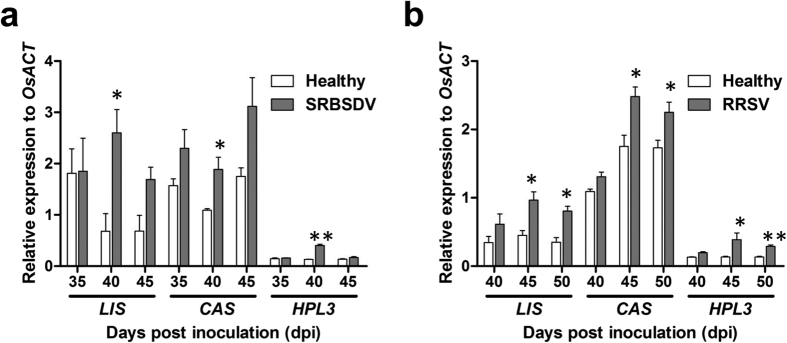
Effect of viral infections on rice volatile biosynthesis. The transcript levels of volatile-biosynthesis genes in SRBSDV-infected (**a**) and RRSV-infected (**b**) plants were compared with those of healthy plants. Total RNA was extracted from leaf sheaths collected at the indicated times and analysed by quantitative reverse transcription polymerase chain reaction. *OsACT* was used as an internal standard. Each data point represents the mean ± standard deviation of three biological replicates. *p < 0.05 and **p < 0.01 for comparisons with healthy plants.

We investigated whether SRBSDV or RRSV influences volatile biosynthesis by analysing the expression levels of *OsLIS, OsCAS*, and *OsHPL3*, which are involved in the production of *S*-linalool, (*E*)-β-caryophyllene, and green leaf volatiles, respectively. The *OsLIS, OsCAS*, and *OsHPL3* transcript abundances were 282.2%, 73.4%, and 209.2% higher, respectively, in SRBSDV-infected plants than in healthy rice plants (t = 3.397, df = 4, p = 0.027; t = 3.023, df = 4, p = 0.039; t = 11.439, df = 4, p < 0.001, respectively) (Fig. 6a).

The expression levels of *OsLIS, OsCAS*, and *OsHPL3* at 45 and 50 dpi were higher in the RRSV-infected rice plants than in the healthy controls. The *OsLIS* transcript abundance was about 2-fold higher in RRSV-infected rice plants than in healthy controls at 45 and 50 dpi (t = 4.327, df = 4, p = 0.012; t = 4.599, df = 4, p = 0.010, respectively). Additionally, the *OsCAS* expression level was about 40% and 30% higher in infected plants than in healthy controls at 45 and 50 dpi (t = 3.334, df = 4, p = 0.029; t = 2.824, df = 4, p = 0.048), respectively. The *OsHPL3* expression level was also 3- and 2-fold higher in RRSV-infected plants than in uninfected plants at 45 and 50 dpi (t = 2.594, df = 4, p = 0.049; t = 6.384, df = 4, p = 0.003), respectively (Fig. 6b). These results suggest that SRBSDV and RRSV infections induce the synthesis of rice volatiles, which may affect the attractiveness of the plants to vector and non-vector planthoppers.

## Discussion

Virus-induced plant responses can influence host-derived cues in ways that positively or negatively affect the attraction of vectors^30^,^31^, which sometimes leads to behavioural changes in the vectors that promote viral transmissions^11^,^32^. To further characterize the interactions among viruses, plants, and vectors, and to complement the work of Wang *et al*.^6^, we compared the attractiveness of SRBSDV- or RRSV-infected plants to virus-free vector and non-vector planthoppers at different disease stages. Our results revealed that viral infections initially decreased, then increased, and finally decreased the attractiveness of rice plants to the analysed planthoppers. The vector and non-vector planthoppers were most attracted to the infected rice plants at 40 days after SRBSDV infection or 45 days after RRSV infection. Additionally, the results of the Y-tube olfactometer tests were similar to those of the free-choice assay, implying the effects of rice on planthopper host-selection preferences were mainly mediated by plant volatiles. Under field conditions, the disease stage is a potential source of variation for plant responses to infection. Blua and Perring reported that the production of *Aphis gossypii* Glover alatae was greater in *Cucurbita pepo* L. plants inoculated with *Zucchini yellow mosaic virus* during the early stages of infection than in healthy plants^33^. Another study concluded that *Myzus persicae* arrestment by the volatiles released from potato plants infected with *Potato leafroll virus* changes as the disease progresses^1^. For example, volatile organic compounds from infected plants are more arresting than the compounds of mock-inoculated controls at 4 and 6 weeks after inoculation, but not at 2, 8, or 10 weeks after inoculation^1^. To more comprehensively characterize the mechanisms underlying vector behavioural responses to SRBSDV or RRSV infections, the disease stages representing all possible field conditions will need to be investigated.

In this study, virus-free WBPH and BPH exhibited different responses to viral infections. For example, at 40 dpi, the WBPHs preferred SRBSDV-infected rice plants to healthy plants. In contrast, there were no differences in the preferences of non-vector BPHs for SRBSDV-infected or healthy plants (Figs 1a and 2a). Regarding RRSV-infected plants, at 45 dpi, the BPHs preferred the infected rice plants over the healthy plants, but no significant difference was observed for the non-vector WBPHs (Figs 1b and 2b). Therefore, in the long term of the evolution, specific plant volatile could be induced by insect vectored virus. Accordingly, the insect have evolved many physiological and behavioural adaptions to recognize those specific external volatiles to help them locate the host plants carrying the virus which is compatible with them. So in this case, investigation of the differences in volatile type and content between SRBSDV and RRSV infected rice plants is warranted.

We observed that the host-selection preferences of the viruliferous planthoppers were not fully consistent between the Y-tube olfactometer and free-choice tests, and the differences varied with disease progression. For example, at 35 days after SRBSDV infection or 20 days after RRSV infection, the SRBSDV-carrying WBPHs and the RRSV-carrying BPHs preferred the healthy plants in the Y-shape olfactometer test (Fig. 1), but preferred the virus-infected plants in the free-choice test (Fig. 2). These results indicate that factors other than olfaction may contribute to the host-selection behaviour of planthoppers. We hypothesized that chlorophyll abundance may influence the host-selection behaviour of the viruliferous planthoppers. By measuring the chlorophyll content in the rice plants, we found that it was higher in SRBSDV or RRSV infected plants than in healthy plants (Fig. 3). The increasing of chlorophyll amount in virus infected plants may due to the swollen chloroplast which we observed in SRBSDV or RRSV infected rice plants by transmission electron microscope (unpublished data). However, our findings indicate that the attractiveness of the SRBSDV- or RRSV-infected plants to WBPHs and BPHs is not associated with chlorophyll accumulation during disease progression. Thus, the chlorophyll in leaves does not have a major role in regulating the host-selection behaviour of planthoppers. The SRBSDV-carrying WBPHs preferred healthy plants and RRSV-infected plants, whereas the RRSV-carrying BPHs preferred healthy plants and SRBSDV-infected plants. This may explain our previous observation that co-infections of RRSV and SRBSDV are common in rice fields, and that the synergism between SRBSDV and RRSV may enhance the ability of infected plants to attract insect vectors^29^. The differences in the preferences of virus-free and viruliferous planthoppers may be due to changes in the olfactory systems of the planthoppers caused by the virus. More research is needed to clarify the complex host-selection behaviour of viruliferous planthoppers.

To determine the relationship between viral load and the attractiveness of infected plants to vector and non-vector rice planthoppers, we continuously monitored the virus titres in infected plants from 15 to 50 dpi. The attractiveness of SRBSDV- or RRSV-infected plants to the planthoppers was positively linearly correlated with their virus titres, and the titre effects were more prominent for virus-free planthoppers than for viruliferous planthoppers. The attractiveness of SRBSDV- or RRSV-infected plants to virus-free WBPHs or BPHs increased as viral contents increased. The highest virus titre was observed at 40 and 45 days for SRBSDV and RRSV, respectively (Fig. 4), which coincided with when the planthoppers were most attracted to the infected plants. The attraction of viruliferous planthoppers to infected plants increased only when the virus titre reached a certain threshold. Additionally, the attractiveness of SRBSDV- or RRSV-infected plants was greater for the viruliferous non-vector planthoppers than for the vector planthoppers. Our results confirm that the amplification of virus particles can induce a large number of viruliferous non-vector planthoppers to localize in infected plants, which contributes to the co-infection of SRBSDV and RRSV. Our previous work observed that the virus titres and ability to take up vectors increase in rice plants co-infected with SRBSDV and RRSV, and the accumulation of plant viruses in vectors is positively associated with transmission efficiency, so the conclusion is that there are positive correlations between viral load and virus acquisition efficiency of vectors^29^. Our recent findings may help to clarify the reasons for the recent prevalence of rice plants co-infected with SRBSDV and RRSV under field conditions.

Recent studies determined that plant defences against phloem-feeding insects mediated by JA, SA, or ET signalling pathways can be inhibited by insect-vectored virus, result in the mutualism between virus and its insect vector^12^,^34^,^35^. For example, a Begomovirus could repress the JA-mediated defences against the whitefly, thereby accelerating its population increase^12^. In this report, we found that the expression of JA, SA, and ET biosynthesis genes were down-regulated by SRBSDV or RRSV infection (Fig. 5), which may also conduct mutualism between the viruses and planthoppers. We concluded that the weakening of defence responses in the SRBSDV- or RRSV-infected plants enable planthoppers to feed on the preferred plants to promote the spread of viruses.

Viral infections may alter plant volatiles in ways that are beneficial or detrimental to the herbivorous viral vectors^36^. In a preliminary choice experiment, we proved that the host-selection behaviour of rice planthoppers was mainly mediated by rice volatiles^6^. Thus, plant volatiles may be important determinants of the composition of insect communities. The *S*-linalool synthase-encoding gene *OsLIS* and the (*E*)-β-caryophyllene synthase-encoding gene *OsCAS* were cloned and analysed^37^,^38^. By silencing these two genes, the inducible *S*-linalool attracted predators and parasitoids as well as chewing herbivores, but repelled the rice brown planthopper^39^. In contrast, the constitutively produced (*E*)-β-caryophyllene attracted both parasitoids and planthoppers, resulting in an increased herbivore load. Meanwhile, Wang *et al*. confirmed that (*E*)-β-caryophyllene functions as a host-locating signal for WBPH^40^. Green leaf volatiles are products of the hydroperoxide lyase branch of the oxylipin pathway, and have important roles during plant defence responses to both insect pests and pathogens^41^. For example, the rice hydroperoxide lyase gene, *OsHPL3*, is up regulated in response to infestations by adult BPH^42^. Additionally, the products of reactions catalysed by OsHPL3 (i.e., mainly (*Z*)-3-hexenal, (*Z*)-3-hexen-1-ol, and (*E*)-2-hexenal) increase the susceptibility of plants to BPH infestations^42^. Therefore, we evaluated whether these specific volatiles influence multi-trophic interactions and insect community composition under field conditions. We quantified the expression levels of some volatile-biosynthesis genes in SRBSDV- or RRSV-infected rice plants. The *OsLIS, OsCAS*, and *OsHPL3* expression levels were considerably higher in infected plants than in healthy plants (Fig. 6). Considered together, these results suggest that up-regulated emission of volatiles in SRBSDV- or RRSV-infected rice plants is linked to increased attraction by planthoppers.

Our results may have implications for the spread of SRBSDV and RRSV within a plant population. Simulation models indicate that vector preferences dependent on infection status can affect the rate of pathogen spread. When infected plants are relatively rare in a plant population, a vector preference for infected plants promotes the spread of infections. In contrast, a preference for healthy plants enhances the spread of disease when infected plants predominate^43^. However, these models do not consider dynamics that depend on disease progression. Our results suggest that numerous WBPHs and BPHs are present in healthy rice plants throughout most of the rice growing period. The healthy plants become their main food source and habitat. Some studies have indicated that the lifespans of female BPHs are shortened in SRBSDV-infected rice plants^44^. The lifespan of WBPHs carrying SRBSDV is less than 15 days at 25 °C^45^. Additionally, the salivary gland cells of BPHs infected by RRSV undergo programmed cell death^46^. These results indicate that infections by SRBSDV or RRSV were not favourable for the growth and development of WBPHs and BPHs, regardless of whether they are carrying a virus. The planthoppers settle in healthy plants for relatively long periods to protect themselves. However, with increasing virus titres, the SRBSDV or RRSV-infected rice plants gradually become more attractive to WBPHs or BPHs. The infected plants were most attractive to the vector and non-vector planthoppers at the highest virus titres. This phenomenon may be explained by the following two points. First, by inhibiting defence responses, the virus-infected plants produce different conditions for the vector and non-vector planthoppers. Second, viral infections significantly affect the biosynthesis of volatiles in rice plants, which is important for attracting vector or non-vector planthoppers. Our future studies will focus on how SRBSDV and RRSV modify rice defence responses and the biosynthesis of volatiles. We will also investigate the effects of specific rice defence signals or volatiles on the attractiveness of plants to planthoppers.

In summary, the dynamics of the relationship between rice planthoppers and infected plants is very complex. Characterizing these dynamics may be relevant for managing SRBSDV and RRSV infections in rice production systems and for understanding the ecology of insect-vectored viruses.

## Methods

### Rice plant, virus, and planthopper materials

The rice plants (*Oryza sativa* L. cv. ‘Nipponbare’) used in this study were grown as previously described^6^,^29^. The SRBSDV and RRSV isolates were maintained on several rice plants grown in an insect-proof greenhouse in our laboratory. The WBPHs and BPHs were reared and propagated on SRBSDV- and RRSV-infected plants, respectively, to become viruliferous. The viruliferous insects were used for subsequent viral inoculations. The SRBSDV- and RRSV- infected rice plants or viruliferous planthoppers were identified by reverse transcription PCR according to the method described by Wang *et al*.^6^.

### Y-tube olfactometer host orientation preference test

The preferences of WBPHs and BPHs for healthy and virus-infected plants were assessed as previously described^6^. The SRBSDV- or RRSV-infected plants were compared with healthy rice plants as two odour sources for the Y-tube olfactometer device. Virus-free WBPH, BPH, viruliferous (SRBSDV-carrying) WBPH, and viruliferous (RRSV-carrying) BPH were analysed at 15, 20, 25, 30, 35, 40, 45, and 50 dpi. Three replicates (ten planthoppers each) were used at each time point. After each test, the whole device was thoroughly washed with distilled water, 90% ethanol, and distilled water again. The device was then heat-dried for later reuse.

### Free-choice host orientation preference test

The free-choice tests were conducted using an improved free-choice device according to a published method^6^. The device was surrounded by transparent polyethylene material (height: 55 cm; diameter: 17 cm), which was connected to a plastic bowl containing 1000 ml nutrient solution. The cover of the plastic bowl was punctured 2 holes 15 cm apart with 2 cm in diameter to enable the insertion of two healthy plants and two infected plants as selection objects. Ten insects were added to the device for free selection of the virus-infected or healthy plants at 15, 20, 25, 30, 35, 40, 45, and 50 dpi. The experiments were conducted with three replicates.

### Determination of rice leaf chlorophyll contents

A SPAD-502 chlorophyll meter [Minolta Camera Co., Osaka, Japan] was used to measure chlorophyll contents of fully expanded leaves from the plant base as well as the middle and lower plant parts at 15, 20, 25, 30, 35, 40, 45, and 50 dpi. Three SPAD readings (dimensionless values; 650/940 nm wavelength transmittance ratio) were taken around the midpoint of each leaf blade, 30 mm from one side of the midrib. Readings for six replicates of SRBSDV- or RRSV-infected and healthy plants were averaged to calculate the mean SPAD readings of each plot.

### Quantitative reverse transcription polymerase chain reaction

The viral genome accumulation level as well as defence and volatile-biosynthesis gene expression levels were analysed by RT-qPCR. Total RNA was extracted from leaf sheaths collected from SRBSDV- or RRSV-infected rice plants at 15, 20, 25, 30, 35, 40, 45, and 50 dpi using Trizol RNAiso Plus (Invitrogen, Boston, MA, USA). The real time PCR was conducted using a Cycler Dice Real Time System TP800 (TaKaRa) and the SYBR Premix Ex Taq II kit (TaKaRa). Primers specific for genome segment 10 of SRBSDV and genome segment 8 of RRSV were used to quantify each virus. The gene encoding rice U6 small nuclear RNA was used as an internal standard for quantifying the relative viral genome accumulation level. *OsACT* (TIGR ID Os03g50885) was used as a reference to quantify the rice defence (*AOS1, ICS*, and *ACS2*) and volatile-biosynthesis (*LIS, CAS*, and *HPL3*) genes. The primers used in this study are listed in Supplementary Table S1.

## Additional Information

**How to cite this article**: Lu, G. *et al*. Virus altered rice attractiveness to planthoppers is mediated by volatiles and related to virus titre and expression of defence and volatile-biosynthesis genes. *Sci. Rep.* **6**, 38581; doi: 10.1038/srep38581 (2016).

**Publisher's note:** Springer Nature remains neutral with regard to jurisdictional claims in published maps and institutional affiliations.

## Supplementary Material

Supplementary Table
