# Supplementary material for: Virus altered rice attractiveness to planthoppers is mediated by volatiles and related to virus titre and expression of defence and volatile-biosynthesis genes
Source: Sci Rep. 2016 Dec 7;6:38581. doi: 10.1038/srep38581 (PMC5141440; doi:10.1038/srep38581)
Supplement: Supplementary Table [file srep38581-s1.doc]

Virus altered rice attractiveness to planthoppers is mediated by volatiles and related to virus titre and expression of defence and volatile-biosynthesis genes

Guanghua Lu*, Tong Zhang*, Yuange He & Guohui Zhou**

**Supplementary Table S1.** Oligonucleotides used in this study.

| **Primer** | **Gene/Application** | **Sequence** |
| --- | --- | --- |
| qACTIN F | *OsACTIN* qRT-PCR | 5'-TGGACAGGTTATCACCATTGGT-3' |
| qACTIN R | *OsACTIN* qRT-PCR | 5'-CCGCAGCTTCCATTCCTATG-3' |
| qAOS1 F | *OsAOS1* qRT-PCR | 5'-CGAGCTCTTCCTCCGATACG-3' |
| qAOS1 R | *OsAOS1* qRT-PCR | 5'-GTCAGAAGGTGGCCTTCTTGAG-3' |
| qICS F | *OsICS* qRT-PCR | 5'-ACCAATTATGTTTCCGATCAATCA-3' |
| qICS R | *OsICS* qRT-PCR | 5'-CGTCGCCTTCTTGGATTTATG-3' |
| qACS2 F | *OsACS2* qRT-PCR | 5'-CTCAGCCCAGCCCATCTCT-3' |
| qACS2 R | *OsACS2* qRT-PCR | 5'-GTAGTAAGGCGCAGCATTAGCA-3' |
| qLIS F | *OsLIS* qRT-PCR | 5'-TTTGATGGATTCATGACAGAGA-3' |
| qLIS R | *OsLIS* qRT-PCR | 5'-CAACAAACTCTGCTGCATTTT-3' |
| qCAS F | *OsCAS* qRT-PCR | 5'-CGCTACGAGATGCTTTTACAAC-3' |
| qCAS R | *OsCAS* qRT-PCR | 5'-CACCGTAGCAGCTACCTGATC-3' |
| qHPL3 F | *OsHPL3* qRT-PCR | 5'-TGGTGCCGACGCAGAAGGTG-3' |
| qHPL3 R | *OsHPL3* qRT-PCR | 5'-GCAGCGGGAAGGAGTGGAGC-3' |
| qU6 F | *OsU6* qRT-PCR | 5'-CGATAAAATTGGAACGATACAGA-3' |
| qU6 R | *OsU6* qRT-PCR | 5'-ATTTGGACCATTTCTCGATTTGT-3' |
| qSR S10 F | *SRBSDV S10* qRT-PCR | 5'-TCATCATTAGCGCGACTAGTTCA-3' |
| qSR S10 R | *SRBSDV S10* qRT-PCR | 5'-CGTCACTCGGCGTCGATAA-3' |
| qRR S8 F | *RRSV S8* qRT-PCR | 5'-GGCTGAGCGTGCGGTTA-3' |
| qRR S8 R | *RRSV S8* qRT-PCR | 5'-TCAGCCTTGATATCGTTGTAGCA-3' |
| dSR S10 F | *S10* SRBSDV detection | 5'-CGCGTCATCTCAAACTACAG -3' |
| dSR S10 R | *S10* SRBSDV detection | 5'-TTTGTCAGCATCTAAAGCGC-3' |
| dRR S8 F | *S8* RRSV detection | 5'-CGCCGTATCTAACGTTCCAG-3' |
| dRR S8 R | *S8* RRSV detection | 5'-TGCCGCGACATAATCAAC-3' |
